# Efficacy and Safety of Coronary Intervention via Distal Transradial Access (dTRA) in Patients with Low Body Mass Index

**DOI:** 10.1155/2022/1901139

**Published:** 2022-08-24

**Authors:** La-Mei Li, Liu-Yan Zhang, Hao-Min Huang, Tao Chen, Feng Li, Gan-Wei Shi, Wen-Hua Li, Jian-Qiang Xiao, Chun Gong, She-Liang Xue, Bo Xu, Jun Gu, Yan-Bin Song, Dan-Dan Shen, Rong-Rong Ji, Gao-Jun Cai

**Affiliations:** Department of Cardiology, Wujin Hospital Affiliated with Jiangsu University, The Wujin Clinical College of Xuzhou Medical University, 2nd Yongning North Road, Changzhou, Jiangsu, China

## Abstract

The study aimed to investigate the efficacy and safety of coronary intervention via distal transradial access (dTRA) in patients with low body mass index (BMI). A total of 67 patients with low BMI who underwent coronary intervention, comprising 29 patients via dTRA and 38 patients via conventional transradial access (cTRA), were retrospectively included. There was no significant difference in the puncture success rate between the two groups (dTRA 96.6%, cTRA 97.4%, *P*=0.846). Compared with the cTRA group, the success rate of one-needle puncture in the dTRA group was lower (51.7% vs. 81.6%, *P*=0.020). The compression haemostasis time in the dTRA group was shorter than that in the cTRA group (*P*  <  0.001). However, the incidence of radial artery occlusion was lower in the dTRA group than in the cTRA group (4.0% *vs*. 33.3%, *P*=0.007). In conclusion, coronary intervention via dTRA was safe and effective in patients with low BMI.

## 1. Introduction

Currently, conventional transradial access (cTRA) has become the default approach for coronary interventional diagnosis and treatment. Compared with the femoral approach, it has lower mortality, a lower risk of massive bleeding, and a decreased incidence of adverse cardiovascular events and vascular complications [1]. However, there are some disadvantages, especially concerning the incidence of radial artery occlusion (RAO). In recent years, coronary interventional diagnosis and treatment via distal transradial access (dTRA) attracted the attention of cardiac intervention experts and gained global popularity as an alternative access route for vascular procedures [2–6]. The success rate of puncture catheterization, the risk of haematoma at the access site, and the occurrence of radial artery spasm are not significantly different between dTRA and cTRA; however, the incidence of RAO is lower, the haemostasis time is shorter, and dTRA has a higher clinical value [7, 8]. Despite these advantages, coronary catheterization via the distal radial artery also exhibits certain disadvantages. For example, because the distal radial artery is smaller and tortuous, the puncture is more difficult, thus resulting in a low puncture success rate and a longer puncture time [9, 10].

Patients with a low body mass index (BMI) may have a smaller distal radial artery diameter and a lower puncture success rate [11]. Some studies have suggested that low BMI may be an independent risk factor for a low success rate of puncture via the distal radial artery [6]. However, there have been few studies on the safety and efficacy of coronary intervention via dTRA (compared with cTRA) in patients with low BMI. Therefore, we conducted a retrospective cohort study to investigate the safety and efficacy of coronary intervention via dTRA in patients with low BMI.

## 2. Subjects and Methods

### 2.1. Patient Population and Study Design

**Figure 1:**
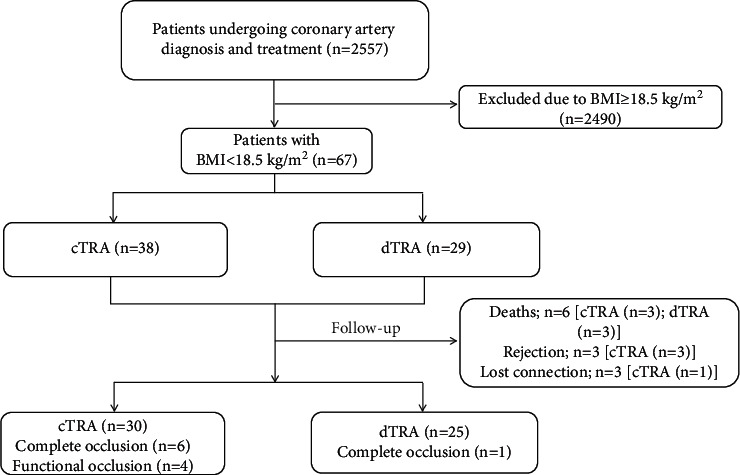
The study flowchart.

A total of 67 low BMI patients who underwent coronary intervention from September 2019 to September 2021 at Wujin Hospital affiliated with Jiangsu University were consecutively included. Thirty-six of the participants were male (53.7%), and the median age was 74 (68–78) years. The numbers of STEMI, NSTEMI, UA, SAP, and non-CAD patients were 13, 7, 12, 19, and 16, respectively (Supplementary Figure 1). The specific screening process is shown in Figure 1. At our centre, the choice of puncture approach was based on the experience of operators. All the puncture operators had rich experience in radial artery puncture. The cTRA group was operated by five cardiologists, all with the experience of radial artery puncture in more than 1,000 cases. The dTRA group was operated by three cardiologists, who had experience of dTRA puncture in more than 100 cases, except for conventional radial artery puncture. According to the preferred interventional route, the patients were divided into the dTRA group (29 patients) and cTRA group (38 patients). RAO was followed up via ultrasound, and the patients were regrouped during follow-up according to the final successful catheterization and interventional procedure approaches. The study was approved by the Ethics Committee of Wujin Hospital affiliated with Jiangsu University (Ethics number: 201938), and all of the patients signed informed consent forms.

## 3. Methods

### 3.1. Data Acquisition

General data, procedure-related data, and follow-up data were recorded. General data included sex; age; height; weight; the history of smoking, drinking, hypertension, diabetes mellitus (DM), coronary artery disease (CAD), cerebral infarction, and hyperlipidaemia; coronary procedures; postprocedural vital signs; and cardiac ultrasound data. Procedure-related data included puncture success rate, puncture time, procedural time, procedural method, procedural category, contrast dosage, radiation exposure time, compression haemostasis time, and complications such as bleeding, haematoma, numbness, hand swelling, and degree of pain. Follow-up data included follow-up time and RAO.

### 3.2. Radial Artery Catheterization Procedure

The surgical methods and procedures of puncture and catheterization were introduced in our previous study [12]. Briefly, the access site in the dTRA group was in the anatomic snuffbox. The patient was asked to place the forearm in a natural vertical position and to hold the thumb under the other four fingers to expose the anatomic snuffbox area. In the cTRA group, the access site was approximately in the proximal three centimetres of the wrist transverse striation. The arm abduction was 70°, and the wrist was overextended, which fully exposed the radial artery. After routine disinfection, local anaesthesia was administered with 2% lidocaine. Subsequently, Seldinger's technique was used to puncture the anatomic snuffbox and the wrist. The puncture was performed with a 20 G puncture needle and a 0.025″ guidewire (Terumo Corporation, Tokyo, Japan). After a successful puncture, an arterial sheath was inserted. After successful cannulation, 3000 U of unfractionated heparin combined with 200 *µ*g of nitroglycerine was administered via the sheath. After the catheterization procedure, a compression device or elastic bandage was used for haemostasis in the cTRA group, and an elastic bandage was used for haemostasis in the dTRA group.

### 3.3. Ultrasound Follow-Up

**Figure 2:**
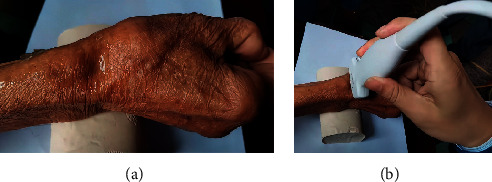
(a) Anatomic snuffbox area in one patient with low body mass index. (b) Ultrasound follow-up operation.

While in a sitting position, the patient placed the ulnar side of the wrist vertically on the examination table, with the hand in a wine-cup shape and relaxed state. The examiner placed the high-frequency probe coated with a coupling agent vertically on the skin at different positions of the anatomical snuffbox. The probe was lightly pressed against the skin to avoid putting pressure on the blood vessels. The radial artery was scanned by using an organized approach. From the position between the middle of the anatomical snuffbox and the first carpometacarpal joint, the middle of the anatomical snuffbox, radial styloid, radial dorsal tuberosity, and the conventional radial artery were detected as anatomical positioning markers. A more detailed assessment of the forearm radial artery can be performed until it is observed to join the brachial artery at the level of the elbow (Figure 2).

### 3.4. Related Definitions

BMI = weight/height^2^, and BMI < 18.5 kg/m^2^ was defined as low BMI [13].

In this study, puncture success was defined as the blood return of the puncture needle and the successful insertion of the radial sheath through the guidewire.

A visual analogue scale (VAS) was used to score the pain of patients with compression haemostasis. A score of 0 points indicated no pain, 1–3 points indicated mild pain that could be tolerated, and 4–6 points indicated tolerable pain and affected sleep. A score of 7–10 indicated intense pain, which was unbearable and affected appetite and sleep [14].

Bleeding was classified by using the Bleeding Academic Research Consortium (BARC) criteria [15], and haematoma was assessed according to the Early Discharge After Transradial Stenting of Coronary Arteries (EASY) classification [16].

Complete occlusion was defined as thrombus formation and no blood flow in the blood vessels, which was indicated via ultrasound. Functional occlusion was defined as no continuous blood flow in the radial artery as indicated by ultrasound, in which the blood flow decreased in a monophasic and slow manner, rather than via complete occlusion [17].

### 3.5. Statistical Analysis

The data analysis was performed with the SPSS 23.0 software. Quantitative data conforming to a normal distribution were expressed as $\bar{x}\pm s$, and an independent sample *t*-test was used for the intergroup comparisons. Measurement data that did not conform to a normal distribution were presented as *M* (*Q*1, *Q*3), and the Wilcoxon test was used for the comparisons between the groups. Qualitative data were expressed as frequency (%), and the Pearson chi-square test was used for the intergroup comparisons. *P* < 0.05 was considered to indicate a statistically significant result.

## 4. Results

### 4.1. Comparison of Baseline Characteristics between the Two Groups

The baseline characteristics are listed in Table 1. There were 29 patients with a median age of 75.0 years in the dTRA group, including 15 males (51.7%). In the cTRA group, there were 21 males (55.3%), and the median age was 73.5 years. There was no significant difference in sex or age between the two groups. In addition, there were no significant differences in BMI, medical history, postprocedure blood pressure, or heart rate compared with the cTRA group. However, the left ventricular end-diastolic diameter (LVEDD) (44.5 vs. 46.0 mm, *P*=0.039) and left ventricular end-systolic diameter (LVESD) (29.5 vs. 32.0 mm, *P*=0.044) in the dTRA group were smaller than those in the cTRA group.

### 4.2. Comparison of the Efficacy between the Two Groups

**Figure 3:**
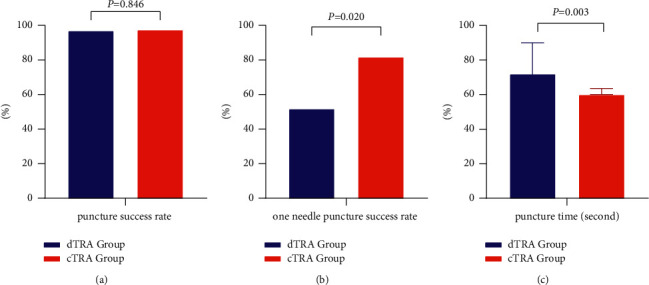
(a) The puncture success rate, (b) the one-needle puncture success rate, and (c) the puncture time between the two groups.

The puncture success rate in the dTRA group was 96.6%, which was not significantly different from that in the cTRA group (97.4%) (*P*=0.846). There was also no significant difference in the puncture success rate of patients undergoing percutaneous coronary intervention (PCI) between the two groups. However, the success rate of one-needle puncture in the dTRA group was significantly lower than that in the cTRA group (51.7% vs. 81.6%, *P*=0.020). Additionally, the puncture time in the dTRA group was longer than that in the cTRA group (72 (60, 90) vs. 60 (60, 63.5) s, *P*=0.003) (Figure 3).

In the cTRA group, one patient could not be punctured in the right conventional radial artery and was switched over to the left conventional radial artery. One patient with puncture failure in the dTRA group was switched to the cTRA group. Ultimately, 28 patients completed the interventional procedure through dTRA, and 39 patients completed the interventional procedure through cTRA. There were no significant differences between the two groups regarding the procedural method, procedural category, contrast dosage, or radiation exposure time. Moreover, a total of 27 patients underwent PCI, of whom 15 patients were implanted with stents. Among them, 9 patients were implanted with one stent, including 4 patients in the cTRA group and 5 patients in the dTRA group. There were 6 patients with ≥2 stents implanted, including 2 in the cTRA group and 4 in the dTRA group. Statistical analysis showed that there was no significant difference in the number of PCI implants between the two groups (*P*=0.228). However, the procedural time in the dTRA group was significantly longer than that in the cTRA group (45 (20, 70) vs. 30 (15, 50) min, *P*=0.043). In addition, the compression haemostasis time in the dTRA group was much shorter than that in the cTRA group (4 (3, 6) vs. 6 (6, 10) h, *P* < 0.001) (Table 2).

### 4.3. Comparison of Safety between the Two Groups

In both groups, bleeding was BARC type II, and haematoma was classified as EASY type I. There was no significant difference in postprocedure bleeding, haematoma, numbness, or hand swelling between the two groups. However, the VAS score in the dTRA group was significantly lower than that in the cTRA group (2 vs. 3, *P* < 0.001) (Table 3). Thirteen patients had ST-segment elevation myocardial infarction, and no hand swelling appeared in either group. There were no significant differences in bleeding, haematoma, numbness, or VAS score between the two subgroups (Supplementary Table 1). Seven patients had non-ST-segment elevation myocardial infarction, and no complications occurred in either group. In addition, there was no significant difference in the VAS scores between the two subgroups (Supplementary Table 2).

### 4.4. Follow-Up Results of Ultrasound

**Figure 4:**
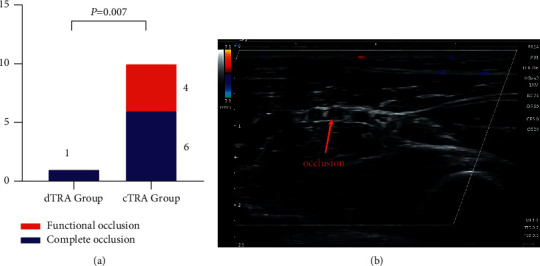
(a) Results of ultrasound follow-up. (b) Ultrasound image of complete radial artery occlusion.

Twelve patients were lost to follow-up. Among them, six patients died (three deaths in the cTRA group and three deaths in the dTRA group), three patients refused follow-up (all in the cTRA group), and three patients lost contact (all in the cTRA group). The ultrasound follow-up time was 13.3 ± 8.2 (months) in the cTRA group (ranging from 31 days to 27 months) and 8.8 ± 5.4 (months) in the dTRA group (ranging from 31 days to 17 months). In the cTRA group, six patients had complete occlusions and four patients had functional occlusions in the proximal radial artery. Interestingly, only one patient had complete occlusion in the dTRA group. The incidence of RAO in the dTRA group was significantly lower than that in the cTRA group (4.0% vs. 33.3%, *P*=0.007) (Figure 4).

## 5. Discussion

To our knowledge, this is the first study to investigate the safety and efficacy of coronary intervention via dTRA in patients with low BMI. This study found that the puncture success rate was similar between the two groups, but the dTRA group had a lower one-needle puncture success rate, a longer puncture time, and a shorter compression haemostasis time than the cTRA group. More importantly, the present study found that the proximal RAO rate in the dTRA group was significantly lower than that in the cTRA group in the low BMI population.

Interventional treatment via dTRA was first reported in 2007 by Pancholy [18], who opened the occluded radial artery via dTRA. In 2017, Kiemeneij [2] published their experiences in 70 cases with coronary intervention through left dTRA. Since then, an increasing number of studies have been performed on different populations. The local anatomical structure of dTRA determines its great advantage in coronary interventional diagnosis and treatment. It can both increase the comfort of patients and reduce the compression time and nursing workload [19, 20]. However, due to the same anatomical characteristics, distal radial artery puncture is more difficult and has a long learning curve [8]. Some studies [9, 10] reported that the success rate in the dTRA group was lower than that in the cTRA group. For example, Lu et al. [21] found that the puncture success rate was 85% in the dTRA group and 100% in the cTRA group (*P* < 0.05). However, an updated meta-analysis [7] found no significant difference in catheterization/puncture failure between the two groups (OR = 1.94, 95% CI: (0.97, 3.86), *P*=0.06).

In a prospective randomized-control study, Eid-lidt G et al. [22] found that the RAO rate in the dTRA group was 0.7% at both 24 h and 30 days after the procedure, which was lower than that in the cTRA group (24 h, OR = 12.8, 95% CI: (1.6, 100.0), *P*=0.002; 30 days, OR = 8.2, 95% CI: (1.0, 67.2), *P*=0.019).

At present, there is no consistent conclusion about the correlation between BMI and radial artery diameter. Dharma et al. [23] found that the conventional radial artery diameter was not associated with BMI. However, Aykan et al. [24] showed that the conventional radial artery diameter was correlated with height (*r* = 0.258, *P* < 0.001), weight (*r* = 0.237, *P* < 0.001), and BMI (*r* = 0.167, *P*=0.013). In a retrospective observational study, Naito et al. [25] found that BMI was not correlated with the diameter of the distal radial artery by using multivariate analysis. However, Norimatsu et al. [11] found that the diameter of the distal radial artery was positively correlated with both body weight (BW) (*r* = 0.248, *P*=0.003) and BMI (*r* = 0.228, *P*=0.007). Although the relationship between BMI and the distal radial artery diameter is still unclear, a previous study [11] found that the distal radial artery diameter may be smaller in patients with low BMI, which increases the difficulty of puncture. Lin Y et al. [6] also found that low BMI was an independent risk factor for the puncture success rate via dTRA, and the cutoff value was 22.04 kg/m^2^ (sensitivity: 76.72%; specificity: 71.43%; area under the ROC curve: 0.72). Patients with low BMI have less subcutaneous tissue in the anatomical snuffbox, and blood vessels are close to the bone plane, which may easily lead to puncture failure, especially when using a trocar puncture. The use of a bare steel needle, which aids in reducing the puncture angle to increase the contact area between the needle and blood vessel, may be beneficial for improving the success rate. Moreover, studies [26, 27] have shown that low BMI is strongly correlated with adverse cardiovascular events and that low BMI increases the risk of bleeding after coronary stenting. However, there are few studies on the safety and efficacy of coronary intervention via dTRA (compared with cTRA) in patients with low BMI. This is the first study to investigate the value of dTRA in coronary intervention in patients with low BMI. Through a retrospective analysis, we found no significant difference in the puncture success rate between the two groups (96.6% vs. 97.4%, *P*=0.846). However, the success rate of one-needle puncture in the dTRA group was lower than that in the cTRA group, and the puncture time was longer than that in the cTRA group. Furthermore, this study found no significant difference in puncture-related complications, such as bleeding and haematoma, between the two groups. However, the postprocedure compression haemostasis time and VAS in the dTRA group were significantly lower than those in the cTRA group, and the patient comfort was higher in the dTRA group.

RAO is a common complication in cardiovascular intervention via the conventional radial artery, and the incidence ranges from 1% to 33% [28]. Several methods have been used to evaluate RAO, such as simple touch, finger blood oxygen saturation test, and Doppler ultrasound. Due to the palmar arch cycle, the previous two methods usually underestimated the incidence of RAO. Ultrasound is more accurate in assessing RAO, as it can identify thrombi and radial artery blood flow and measure the intima thickness of the radial artery [29]. Some studies [7] have been conducted to evaluate the advantage of dTRA in reducing the incidence of RAO. In a single-centre prospective study, Lin et al. [6] found that the incidence of RAO in the dTRA group was significantly lower than that in the cTRA group (1.56% vs. 3.78%, *P*=0.033). Additionally, some studies [30] have shown that lower BMI is one of the predictors of RAO. However, there are few studies on whether dTRA can reduce the incidence of RAO in patients with low BMI. Our study also found that among the low BMI population, there were six complete occlusion patients and four functional occlusion patients in the cTRA group, and the total RAO incidence was 33.3%, which was much higher than that in other populations (5.6%) [22]. However, there was only one patient with complete RAO in the dTRA group, which was a much lower incidence than that in the cTRA group (4.0% *vs.* 33.3%, *P*=0.007). Notably, this patient had a history of coronary intervention via cTRA, and injury during a previous procedure may be one of the factors that contribute to RAO.

There were several limitations of this study. First, this study was a single-centre, retrospective cohort study with a small sample size, which may have resulted in certain deviations. Second, ultrasound examination was not performed on all of the patients before the procedure; thus, it was not known whether the proximal radial artery had lesions before puncture. In addition, dTRA punctures performed by experienced physicians who have overcome the learning curve may overestimate the success rate of dTRA punctures in patients with low BMI.

In conclusion, coronary intervention via dTRA was safe and effective in patients with low BMI, with a short compression haemostasis time, increased comfort for patients, and significantly reduced incidence of RAO.

## Figures and Tables

**Table 1:** Comparison of baseline characteristics between the two groups.

Characteristics	dTRA (*n* = 29)	cTRA (*n* = 38)	*P*
Male (*n* (%))	15 (51.7)	21 (55.3)	0.773
Age (M/(P25, P75), years)	75.0 (67.0, 78.5)	73.5 (69.0, 78.3)	0.820
BMI (M/(P25, P75), kg/m^2^)	17.6 (16.5, 18.2)	17.4 (16.7, 18.0)	0.621
Smoking (*n* (%))	9 (31.0)	9 (23.7)	0.501
Drinking (*n* (%))	2 (6.9)	1 (2.6)	0.403
Hypertension (*n* (%))	14 (48.3)	13 (34.2)	0.245
DM (*n* (%))	5 (17.2)	6 (15.8)	0.874
CAD (*n* (%))	10 (34.5)	8 (21.1)	0.219
Cerebral infarction (*n* (%))	2 (6.9)	0 (0.0)	0.100
Hyperlipidaemia (*n* (%))	1 (3.4)	0 (0.0)	0.249
Several procedures^*∗*^ (*n* (%))	6 (20.7)	4 (10.5)	0.247
Postprocedural systolic pressure (($\bar{x}\pm s$), mmHg)	132.7 ± 24.3	131.7 ± 22.8	0.861
Postprocedural diastolic pressure (($\bar{x}\pm s$), mmHg)	74.6 ± 11.0	76.4 ± 11.5	0.503
Postprocedural heart rate (M/(P25, P75), bpm)	72.0 (68.0, 81.0)	74.0 (65.5, 86.0)	0.746
LVEDD (M/(P25, P75), mm)	44.5 (42.5, 46.0)	46.0 (42.5, 50.0)	0.039
LVSD (M/(P25, P75), mm)	29.5 (26.8, 31.3)	32.0 (28.0, 35.0)	0.044
EF (M/(P25, P75), %)	62.0 (57.0, 67.0)	60.5 (46.5, 66.5)	0.244

^*∗*^At least two times of ipsilateral interventional procedures; BMI, body mass index; DM, diabetes mellitus; CAD, coronary artery disease; LVEDD, left ventricular end-diastolic diameter; LVESD, left ventricular end-systolic diameter; EF, ejection fraction.

**Table 2:** Comparison of the efficacy between the two groups.

Characteristic	dTRA (*n* = 28)	cTRA (*n* = 39)	*P*
Procedural time (M/(P25, P75), min)	45 (20, 70)	30 (15, 50)	0.043
Procedural method (*n* (%))			
CAG	15 (53.6)	25 (64.1)	0.386
PCI	13 (46.4)	14 (35.9)	
Procedural category (*n* (%))			
Emergency	6 (21.4)	9 (23.1)	0.873
Routine	22 (78.6)	30 (76.9)	
Contrast dosage (M/(P25, P75), ml)	100 (50, 150)	60 (50, 100)	0.113
Radiation exposure time (M/(P25, P75), min)	9.5 (3.1, 15.1)	3.3 (1.7, 11.9)	0.181
Compression haemostasis time (M/(P25, P75), h)	4 (3, 6)	6 (6, 10)	<0.001

CAG, coronary angiography; PCI, percutaneous coronary intervention.

**Table 3:** Comparison of the safety between the two groups.

Characteristics	dTRA (*n* = 28)	cTRA (*n* = 39)	*P*
Bleeding (BARC II) (*n* (%))	3 (10.7)	7 (17.9)	0.388
Haematoma (EASY I) (*n* (%))	1 (3.6)	0 (0.0)	0.240
Numbness (*n* (%))	0 (0.0)	2 (5.1)	0.224
Hand swelling (*n* (%))	0 (0.0)	1 (2.6)	0.393
VAS (M/(P25, P75))	2 (2, 3)	3 (3, 4.5)	<0.001

VAS, visual analogue scale; BARC, Bleeding Academic Research Consortium; EASY, Early Discharge After Transradial Stenting of Coronary Arteries.

## Data Availability

The datasets used and/or analyzed during the current study are available from the corresponding author upon request.

## Supplementary Materials

Supplementary Materials Supplementary Figure 1: The number of STEMI, NSTEMI, UA, SAP, and non-CAD patients between the two groups. Supplementary Table 1: Comparison of the safety in patients of STEMI between the two subgroups. Supplementary Table 2: Comparison of the safety in patients of NSTEMI between the two subgroups.
