# Supplementary material for: Efficacy and Safety of Coronary Intervention via Distal Transradial Access (dTRA) in Patients with Low Body Mass Index
Source: J Interv Cardiol. 2022 Aug 24;2022:1901139. doi: 10.1155/2022/1901139 (PMC9433246; doi:10.1155/2022/1901139)
Supplement: Supplementary Materials — Supplementary Figure 1: The number of STEMI, NSTEMI, UA, SAP, and non-CAD patients between the two groups. Supplementary Table 1: Comparison of the safety in patients of STEMI between the two subgroups. Supplementary Table 2: Comparison of the safety in patients of NSTEMI between the two subgroups. [file 1901139.f1.zip › Supplementary Figures.doc]

**Supplementary Figure 1** The number of STEMI, NSTEMI, UA, SAP and non-CAD patients between the two groups.

**(A)** cTRA group. **(B)** dTRA group.

Non-CAD, non coronary artery disease; STEMI, ST-segment elevation myocardiol infarction; NSTEMI, Non-ST-segment elevation myocardiol infarction; UA, unstable angina pectoris; SAP, stable angina pectoris.
