# Supplementary material for: Efficacy and Safety of Coronary Intervention via Distal Transradial Access (dTRA) in Patients with Low Body Mass Index
Source: J Interv Cardiol. 2022 Aug 24;2022:1901139. doi: 10.1155/2022/1901139 (PMC9433246; doi:10.1155/2022/1901139)
Supplement: Supplementary Materials — Supplementary Figure 1: The number of STEMI, NSTEMI, UA, SAP, and non-CAD patients between the two groups. Supplementary Table 1: Comparison of the safety in patients of STEMI between the two subgroups. Supplementary Table 2: Comparison of the safety in patients of NSTEMI between the two subgroups. [file 1901139.f1.zip › Supplementary Table 1.doc]

**Supplementary Table 1: Comparison of the safety in patients of STEMI between the two subgroups**

| **Characteristic** | **dTRA (n=4)** | **cTRA (n=9)** | ***P*** |
| --- | --- | --- | --- |
| Bleeding (BARC II) [n (%)] | 1 (25) | 2 (22.2) | 1.0 |
| Haematoma (EASY I) [n (%)] | 1 (25) | 0 (0.0) | 0.308 |
| Numbness [n (%)] | 0 (0.0) | 1 (11.2) | 1.0 |
| Hand swelling [n (%)] | 0 (0.0) | 0 (0.0) |  |
| VAS (‾x ± s ) | 2.75±0.96 | 3.78±2.11 | 0.379 |
